# Navigating barriers and facilitators to lifestyle changes after bariatric surgery among Emirati adolescents: a qualitative study

**DOI:** 10.3389/fpubh.2025.1702340

**Published:** 2025-11-26

**Authors:** Baraa A. Alries, Efstathia Papada, Hazem Al Momani, Amjad H. Jarrar, Ayesha S. Al Dhaheri, Leila Cheikh Ismail, Lily Stojanovska, Habiba I. Ali

**Affiliations:** 1 Department of Nutrition and Health, College of Medicine and Health Sciences, United Arab Emirates University, Al Ain, United Arab Emirates; 2 Division of Medicine, University College London, London, United Kingdom; 3 Weight Management Unit, NMC Royal Khalifa Hospital, Abu Dhabi, United Arab Emirates; 4 Department of Clinical Nutrition and Dietetics, College of Health Sciences, University of Sharjah, Sharjah, United Arab Emirates; 5 Nuffield Department of Women’s & Reproductive Health, University of Oxford, Oxford, United Kingdom; 6 Institute for Health and Sport, Victoria University, Melbourne, VIC, Australia

**Keywords:** adolescent, bariatric surgery, qualitative, exercise, social environment, diet, United Arab Emirates

## Abstract

Adolescent obesity is a major public health concern, with bariatric surgery emerging as an effective treatment option. However, little is known about adolescents’ long-term adherence to healthy behaviors after surgery, especially in non-Western settings. This qualitative interview study explored lifestyle challenges faced and enablers experienced by adolescents who had undergone bariatric surgery at least one year before the interview, addressing a research gap. Semi-structured interviews were conducted with 15 adolescents (12–19 years) who had undergone bariatric surgery (sleeve gastrectomy: 86.6%; Roux-en-Y gastric bypass: 13.2%) more than a year ago, using the social–ecological model (SEM) to guide data collection and interpretation. The interviews revealed five key themes: Motivations for bariatric surgery, post-surgery barriers to regular physical activity, enablers for overcoming post-surgery challenges, post-bariatric surgery lifestyle change strategies, and suggestions for overcoming post-surgery challenges. Barriers to regular physical activity included family and academic responsibilities, as well as environmental, psychological, and medical factors. Health challenges were more difficult to manage than food or physical activity. Support from healthcare teams played a crucial role in overcoming post-bariatric surgery challenges, especially those related to diet and medication adherence. Participants recommended adapting lifestyle changes and consulting health professionals to overcome post-bariatric surgery challenges.

## 1 Introduction

According to the World Health Organization (WHO), the prevalence of overweight and obesity among 5–19-year-old children and adolescents has increased dramatically from 8% in 1990 to 20% in 2022 (1). A Sematic Review and Meta-Analysis of data from 154 countries found high prevalence of overweight and obesity among children and adolescent (2). The Middle East has the second-highest obesity rates globally, with 75% of the population living with obesity or being overweight, mainly within the Gulf states (3). Moreover, the prevalence of obesity among children and adolescents is higher in this region than the global average, with rates ranging between 5 and 18% (3). The increasing prevalence of obesity in the Arab Gulf region has been attributed to external factors such as shifts in diet, socioeconomic changes, demographics, and urbanization, along with factors such as genetic predisposition (2).

Previous studies conducted in the UAE reported high prevalence of overweight and obesity among adolescents (4, 5). Among 38,813 adolescents aged 1–18 years who attended community -based healthcare facilities in Dubai between 2018 and 2023, the prevalence of obesity and severe obesity were 10.8 and 6.2%, respectively (6). Moreover, a large-scale epidemiological study (*n* = 44,942) conducted in Ras Al-Khaimah, the United Arab Emirates (UAE), revealed a steady increase in obesity among individuals aged 3–18, with 2.36 and 0.28% becoming obese and extremely obese, respectively, annually (7), a serious public health concern.

Adolescents living with obesity are at risk of continuing to be obese into their adulthood, and developing related health conditions (8, 9). Type 2 diabetes mellitus, hypertension, insulin resistance, and glucose intolerance are more likely to appear among children or adolescents living with obesity (9, 10). In addition, adolescents with obesity face a variety of emotional challenges, such as low self-esteem and self-worth, poor educational outcomes, and difficulties in building and supporting peer relationships (11). Furthermore, adolescents with morbid obesity often experience bullying and victimization due to societal stigma, appearance-based discrimination, and negative weight-related stereotypes (12). These factors lead to social exclusion and being targeted (12).

The emergence of these comorbidities at young ages increases their medical needs and healthcare costs (13). Therefore, obesity among youth is a serious problem that has prompted revisions of existing treatment protocols, which have considered the introduction of surgical treatment (14).

Treatment for adolescent obesity begins with behavioral management, including encouraging healthier eating habits, increasing physical activity levels, and promoting long-term maintenance of these changes (15). Moreover, pharmacotherapy treatment and bariatric surgery can be classified as a type of treatment for adolescent obesity (15). In particular, bariatric surgery is encouraged for adolescents with severe obesity (16, 17).

The indications for adolescent metabolic and bariatric surgery include body mass index (BMI) criteria: a BMI ≥ 35 kg/m^2^ or 120% of the 95th percentile with clinically significant comorbid conditions, including obstructive sleep apnea, type 2 diabetes, idiopathic intracranial hypertension, nonalcoholic steatohepatitis, Blount’s disease, slipped capital femoral epiphysis, gastroesophageal reflux disease, or hypertension or a BMI ≥ 40 kg/m^2^ or 140% of the 95th percentile (whichever is lower) (18). A multidisciplinary team should evaluate patient and family readiness to assess the patient’s and their family’s ability and motivation to adhere to pre- and post-operative treatment plans, including the consistent use of micronutrient supplements (18).

Procedures such as sleeve gastrectomy and gastric bypass have demonstrated encouraging outcomes in addressing severe adolescent obesity (19). These surgical interventions have led to a substantial weight loss of 23–28% after 1 year and 16–22% after 5 years when compared with individuals who did not undergo surgery (19).

After bariatric surgery, lifestyle changes, particularly dietary habits and physical activity, are crucial for reducing obesity rates (20). In the first year post-surgery, adolescents follow a restricted diet due to a reduced stomach size, impacting hormone regulation and intake (21). After the first year, their diet becomes more diverse (21), highlighting the need for research into changing dietary habits for evaluating long-term outcomes and identifying unmet needs.

The Social Ecology Model (SEM) is a framework that is frequently used in public health research and practice (22). Building on Bronfenbrenner’s multilevel framework, McLeroy et al. proposed the Social Ecological Model (SEM) consisting of five levels influencing health behavior: intrapersonal (e.g., beliefs and behaviors), interpersonal (e.g., family and peer influences), organizational (e.g., institutional policies), community (e.g., local resources and programs), and public policy (e.g., government regulations on health and education, such as access to healthy foods, and physical education requirements in schools) (23). Accordingly, these five levels can have an impact on adolescent behaviors (24).

The SEM framework is relevant for exploring the complex determinants of adolescent health behaviors following bariatric surgery. In particular, few studies that examined the challenge of determining whether adolescents readily adopt changes in diet and physical activity 1 year after bariatric surgery have been conducted worldwide, highlighting a notable research gap (20). Similarly, there are no studies conducted in the UAE on this aspect. Thus, the main objective of this study was to explore the perspectives of adolescents at least 1 year after bariatric surgery on their barriers and facilitators related to diet and physical activity.

## 2 Methods

### 2.1 Design and participants

This study employed a qualitative design that included in-depth individual interviews with adolescents who had undergone bariatric surgery to explore barriers and facilitators related to diet and physical activity 1 year after bariatric surgery. A qualitative interview was deemed a suitable method to explore perspectives. This study was conducted in a major hospital in Abu Dhabi specializing in maternal and child healthcare.

The inclusion criteria were adolescent males and females aged between 12 and 19 years who had undergone bariatric surgery at least 1 year before the date of the interview. Adolescents who underwent laparoscopic sleeve gastrectomy, laparoscopic mini gastric bypass, and laparoscopic Roux-en-Y gastric bypass with or without comorbidities were included in the study. Those who had gastric balloon insertion or gastric plication, were younger than 12 years old or older than 19 years old, or became pregnant after surgery, were excluded from the study. According to the WHO, adolescents are typically defined as individuals aged 10–19. This age group covers the transition from childhood to adulthood and includes early, middle, and late adolescence (25).

### 2.2 Data collection

**Figure 1:**
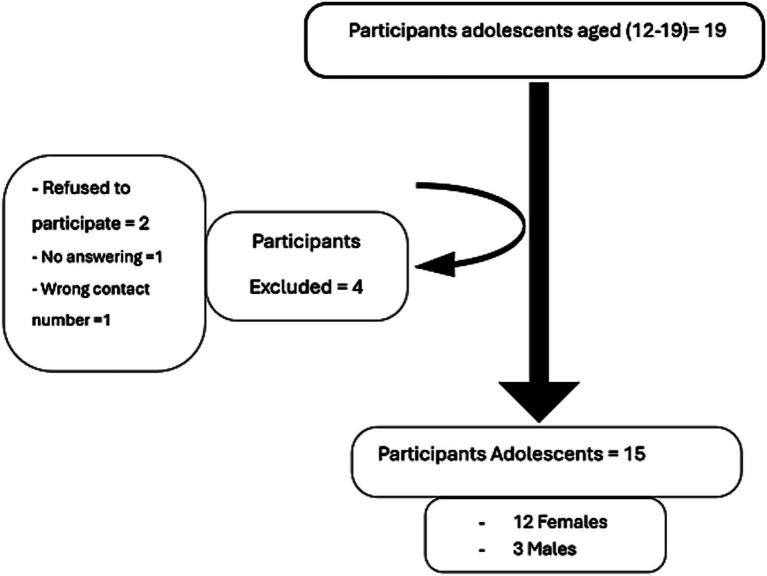
Participant flow chart.

The data for this study were collected from June to July 2022. All adolescent males and females who had undergone bariatric surgery in a major hospital for women in Abu Dhabi between June 2018 and June 2020 were contacted and invited to participate in the study. This time frame was chosen to ensure that the study would include participants for whom more than 1 year had elapsed since surgery. The study identified 19 adolescents who underwent bariatric surgery at least 1 year before the study from the hospital’s medical records, and four were excluded. Two patients refused to participate, one did not respond to calls, and one had an incorrect contact number. The remaining 15 adolescent patients participated in the study (Figure 1).

The participants’ medical records were used to obtain information about the type and date of surgery, anthropometric measurements (weight, height, BMI), and medical history, encompassing conditions such as diabetes, hypertension, hypothyroidism, and sleep apnea. The CDC growth chart BMI for age was used to classify the participants’ weight status as underweight, overweight, or obese (26).

Before the data collection, the participants were given information sheets and consent forms to be signed by their parents/guardians. They also received a verbal outline of the project over the phone immediately before the study to ensure they understood the information. The letter sheet included information about the study’s title, the aim of the analysis, its benefits, what participants should do, and that attendance was entirely voluntary. This information letter was sent via WhatsApp to participants (and their parents if the participants were under 19 years old) and was collected after obtaining their signatures.

This information letter was sent via WhatsApp to participants (and their parents if the participants were under 19 years old) and was collected after obtaining their signatures.

### 2.3 Interview discussion guide

The interview discussion guide contained 12 questions, and its development was guided by the Socio-ecological Model (SEM) (27, 28). It focused on three key areas: (1) Personal level: reasons for the motivation to undergo bariatric surgery, physical activity, and dietary habits after bariatric surgery (four key questions); (2) challenges and facilitators (personal and interpersonal): four key questions; and (3) institutional level (healthcare team) and community levels (four key questions). Additional probes were used to obtain further information as necessary. The interview discussion guide is available as Supplementary material S1.

The research team, consisting of a dietitian (first author) and a university faculty member with expertise in qualitative research, developed the interview discussion guide. The questions aimed to elicit adolescent patients’ experiences at least 1 year after bariatric surgery, on motivators for bariatric surgery, lifestyle challenges, and facilitators after the surgery.

The interview guide was translated into Arabic and underwent content validation before data collection. This process included a review by two bilingual Arabic and English research team members to ensure that the questions aligned with each level of the SEM. A pretest for the interview was conducted with three participants before the formal interviews.

### 2.4 Interviews

A female interviewer conducted semi-structured, individual interviews with the 15 adolescents using an interview discussion guide designed to guide the interview process (28). The interviewer was a native Arabic-speaking graduate student and the study’s first author. She attended a training session on qualitative research interviewing conducted by the last author. The interview was not associated with any of the study participants. Her only prior communication with the participants was through WhatsApp, inviting them to the interviews. The interviews were conducted in Arabic because it is the preferred language for the participants. The interviews were conducted by phone in the presence and with the permission of the parent/guardian of the participant and were audio recorded. Throughout the interviews the interviewer considered the interview guide and her own conduct to minimize bias. The interviews stopped when all 15 participants who agreed to participate in the study were interviewed. The duration of each interview was 15 to 20 min.

### 2.5 Data analysis

Because the interviews were collected in Arabic, the interviewer translated the interview transcripts into English so that the NVIVO software could be used for text-based analysis. Data were analyzed using the inductive thematic method (29). The transcripts were checked for completeness and against the original Arabic recordings. The interview transcripts were imported into NVIVO 12 software. The data were analyzed by reviewing the interviews to establish the initial codes and categories (29, 30). To facilitate the categorization of the initial concepts, the information from each interview was entered into the Nvivo12 software. Based on this first analysis, the interview questions were improved with additional examinations using the qualitative research technique (31). The constant comparison method was used to identify the initial codes, create categories, conduct a systematic comparison, and group the interview responses into themes (26, 30). The transcripts were read several times to become acquainted with the interview data to recognize the starting codes. The codes were continuously compared, divided into categories and themes, and then further divided into sub-themes. The first author performed the initial coding, which was reviewed by the last author, who has experience with qualitative research. The emerging themes and sub-themes from the interviews were categorized into individual, interpersonal, institutional (healthcare), and community levels according to the SEM (23, 29). Descriptive statistics (percentages, means, and standard deviations) were performed using Microsoft Excel to analyze participant demographic information.

### 2.6 Quality assurance

Several methods were used to increase the quality of the study findings. An independent reviewer who is fluent in Arabic and English verified the consistency of the Arabic transcriptions and their translations into English. In addition, the translated transcript and 15 recorded interviews were sent to an external reviewer, who confirmed that the English translations matched the Arabic recordings. The Results section includes participant references that support the main themes and sub-themes that were developed. These quotes are supplied to increase the worthiness of derived themes and sub-themes. The interviews were audio recorded and translated into Arabic verbatim. An external reviewer checked both the Arabic transcription from the recordings and the English translation of the Arabic transcripts. The study adhered to the Consolidated Criteria for Reporting Qualitative Research (COREQ) checklist (32) to maintain transparency and comprehensive reporting of the qualitative research process (Supplementary material S2).

## 3 Results

### 3.1 Participant characteristics

Table 1 provides an overview of the participants’ demographic characteristics. A total of 15 individual interviews were conducted, with most (80%) being females. The average age of the participants was 17.6 ± 1.75 years. Sleeve gastrectomy was the most performed surgical procedure, representing 86.6% of all surgeries. Most participants had undergone surgery more than 25 months before the interview (73.3%), and most had no significant medical history (86.6%).

**Table 1:** Sample demographic characteristics (*n* = 15).

Characteristic	*n* (%)	Mean ± SD	
		Male	Female
Male	3 (20)		
Female	12 (80)		
Age		17.3 ± 2.6	17.7 ± 1.5
Height (m)		181.8 ± 2.4	157.7 ± 6.6
Pre-surgery weight (kg)		174.4 ± 17.8	106.3 ± 18.2
% of >95% BMI for age^*^	15 (100)	52.8 ± 6.1	42.5 ± 5.2
Insurance cover	15 (100)		
Sleeve gastrectomy	13 (86.6)		
Roux-n-Y-gastric bypass	2 (13.3)		
12–18 months post-surgery	2 (13.3)		
19–24 months post-surgery	2 (13.3)		
>25 months post-surgery	11 (73.3)		
Medical history			
Obstructive sleep apnea	1 (6)		
Hypothyroidism	1 (6)		
Type 2 diabetes, primary hypertension, hypothyroidism	1 (6)		
None	13 (86.6)		
Insurance covered (surgery, consultations, and follow-ups visits)	15 (100)		

* Based on the CDC2000 growth charts.

### 3.2 Themes

Five primary themes emerged from the interviews. The first theme, “Motivations for bariatric surgery,” consisted of internal and external motivator sub-themes. The second theme was “Post-surgery challenges,” which included barriers to physical activity and other challenges. The third theme was “Enablers for overcoming post-surgery challenges,” which entailed support from the healthcare team and access to healthy food options in schools. The fourth theme was “Post-bariatric surgery lifestyle change strategies,” which focused on both successful post-surgery adaptation of self-management strategies and limited adaptation to lifestyle changes due to certain challenges. Finally, the fifth theme focused on “Suggestions” for overcoming post-surgery challenges (Table 2).

**Table 2:** Main themes and subthemes.

Theme	Sub-theme
Theme 1: motivations for bariatric surgery	Internal motivators External motivators
Theme 2: post-surgery challenges	Barriers to physical activity Other challenges
Theme 3: enablers for overcoming post-surgery challenges	Healthcare support Access to healthy food options in schools
Theme 4: post-bariatric surgery lifestyle change strategies	Successful adaptation to lifestyle Changes Challenges in adaptation to lifestyle changes
Theme 5: suggestions for overcoming post-surgery challenges	Healthcare team support Self-management behaviors

#### Theme 1: motivations for bariatric surgery

##### Sub-theme 1: internal motivators

Weight gain due to eating more and less physical activity during COVID-19 was also a motivator to undergo bariatric surgery.

“*During the COVID-19 pandemic, I chose to eat more while having less physical activity, primarily due to being engaged in online studying. Controlling my appetite became a challenge. Despite my parents initially disagreeing about the surgery, I persuaded them to consult with a doctor and make the decision together*” (Participant #5, F, 15 years).

Another participant said,

“*Actually, I tried many types of diet, and I did exercise. I was losing weight, but then I regained the weight I had lost. Also, I was consuming a large meal. Then, I saw my brother do the surgery. He was happy with his experience, so I made the decision*.” (#7, M, 19 years).

##### Sub-theme 2: external motivators

This study identified various motivators for seeking bariatric surgery, leading to the classification of two sub-themes: internal and external motivators. External factors were the primary motivators, whereas internal motivators were less common. Of the 15 participants, only three had a past medical condition such as type 2 diabetes, primary hypertension, or sleep apnea, which served as internal motivators for their decision to undergo surgery.

Most participants reported parents, family members, a friend with previous bariatric surgery experience, or a healthcare provider (surgeon) as their motivator for the surgery

“*I did a lot of meal plans, but I felt that it was not working. My friends and family encouraged me to do the surgery to lose weight*” (Participant #13, F, 19 years).

Another reason raised by the participants was related to motivation from family and doctors due to medical conditions, such as type 2 diabetes and hypothyroidism.

“*I had tried many diets before, but they were useless. Also, I had diabetes. My family and doctor motivated me to do the surgery because losing weight would help the diabetes disappear*” (Participant #9, F, 19 years).

#### Theme 2: post-surgery challenges

The participants mentioned several post-surgery challenges, which were further categorized into two sub-themes: Barriers to physical activity and other challenges.

##### Sub-theme 1: barriers to physical activity

Time constraints due to academic responsibilities were a significant barrier to physical activity:

“*Immediately, post-surgery, I was doing regular physical activities, but after a few months, I had stopped due to my studies. In my free time, sometimes, I swim just for fun*” (P#5, F, 15 years).

Another participant stated:

*“Due to my school and academic commitments, I couldn’t engage in regular physical activities. I only managed to walk 2–3 times a week, which was not consistent—some weeks I did, while others I did not*.” (P#10, M, 19 years)

Other physical activity barriers included a lack of access to safe exercise facilities and a limited social support network:

“*I don’t do any regular physical activity. I plan to start in the gym, but the problem is that I am alone and don’t know how to use the machines there*” (P#11, F, 16 years).

Lack of motivation negatively impacted physical activity engagement:

“*I engaged in consistent physical activity for a year after the surgery, but eventually, I discontinued it due to feeling bored*” (P#13, F, 19 years).

Medical challenges, such as chronic health conditions or physical disabilities that limit mobility or cause pain during physical activity, also negatively impacted regular physical activity.

“*Yes, I was doing regular physical activity; then I stopped because my iron level had decreased, and I have low blood pressure when sleeping, sitting, or swimming*” (P#12, F, 19 years).

##### Sub-theme 2: other post-surgery challenges

After undergoing bariatric surgery, the participants encountered a range of challenges, such as feeling dizzy or lethargic, and reported low water intake and hair loss. Losing weight, especially after 1 year of surgery, was another challenge that some of the adolescent participants encountered. Furthermore, some participants experienced challenges related to their health status, such as stomach ulcers and hypoglycemia, as well as a lack of energy. One participant commented:

“*I faced no physical activity or food challenges; the only challenges were hair loss, from the physical side, dizziness, and lethargy*” (P#11, F, 16 years).

Another participant stated:

“*I did not experience any challenges after the surgery; the only issue was hair loss*” (P#5, F, 15 years).

#### Theme 3: enablers for overcoming post-surgery challenges

Most participants did not mention food or physical activity as their primary post-surgery challenges. They emphasized various enablers that aided them in overcoming their challenges due to the support they received from their healthcare team. When asked about the foods available in the academic or work settings, most participants indicated the availability of healthy food options:

“*All food choices that I need are available and easy to get*” (Participant #5, F, 15 years).

However, one of the participants felt that the food in the university residential campus is not healthy for adolescents after bariatric surgery.

“*Not all the food choices that I need are available and easy to get, especially since I am living in a university hostel. But I bought the food and cooked*” (Participant #6, F, 19 years).

Participants preferred to seek healthcare advice through doctor consultations and followed the medical instructions for taking medications and vitamins and receiving intravenous (IV) fluids. One of the participants stated:

“*I was going to the hospital to take IV fluids and other vitamins or medications*” (P#11, F, 16 years).

#### Theme 4: post-bariatric surgery lifestyle change strategies

This theme was categorized into two sub-themes: successful adaptation to lifestyle changes and challenges in adaptation to lifestyle changes.

##### Sub-theme: successful adaptation to lifestyle changes

After bariatric surgery, eating habits and regular exercise are the main elements requiring change. However, how the participants adapted differed. Some participants followed the self-support strategy in adapting to lifestyle changes, such as making healthy food choices, eating less, taking medications, and engaging in regular exercise. Moreover, some participants changed the amount of food they ate and tried to make healthy food choices:

“*Yes, it is changing to become better; I increased my water intake, less amount of food, light food (low fat), and very little sugar*” (Participant #14, F, 19 years)

Another participant said:

“*Yes, it is changing to become better. I am choosing healthier choices. For example, if I have a few healthy food choices, I try to choose the healthier one between them. Also, my food choices depend on food tolerance; for example, I cannot tolerate rice and sweets, so my consumption is less”* (Participant # 3, F, 19 years).

Meanwhile, with regard to the importance of regular physical activity, one of the participants said:

“It is necessary to do physical activities; I believe that regular physical activity is playing an important role in losing weight after surgery” (P#2, M, 14 years).

##### Sub-theme challenges in adaptation to lifestyle changes

A few of the participants stated that they were unable to adapt to the required lifestyle change following bariatric surgery. According to one of the participants, she still eats the same as before the surgery:

“*Home food is mostly prepared light [healthy…]. My food choices are still the same as before surgery, such as full-fat dairy products, refined sugar, and white or brown bread, depending on what is available*” (P#15, F, 19 years).

Another participant said:

“*I dislike doing exercise*” (Participant #5, F, 15 years).

#### Theme 5: suggestions to overcome post-surgery lifestyle challenges

Suggestions for supporting adolescents in managing post-bariatric surgery challenges related to food, exercise, and weight loss were categorized into two sub-themes: healthcare team support and self-management behaviors. Most participants believed that following healthcare team instructions, especially those of the consultant, played an essential role in overcoming challenges, whereas others thought that the dietitian had a more important role.

*“The role of the dietitian is essential because it is important to know what to eat and how to eat post-surgery*” (P#3, F, age 19 years).

Some participants suggested that eating healthy food and exercising regularly played an essential role in overcoming post-bariatric surgery challenges. In addition, it was crucial to increase awareness of healthy food and general health.

“*Exercise and eat healthily*” (P#12, F, 19 years).

When asked if participants believe there is a need to develop new programs to facilitate weight loss maintenance after bariatric surgery for adolescents in the hospital, they stated that the current healthcare team members (doctor, dietitian, and nurse) are sufficient. Thus, there was no need to develop any new program in the hospital:

“*All is good, the healthcare team is complete*” (P#15, F, 19 years).

On the other hand, most participants expressed interest in joining a community-based program for adolescents aimed at maintaining weight loss after surgery:

*“Yes, I am willing to join it to take care of my health.”* (Participant #3, F, 19)

## 4 Discussion

This qualitative study used the social-ecological model (SEM) to explore the factors influencing dietary and physical activity behaviors, and related barriers and facilitators of 15 adolescents aged 12–19 years old in the UAE, after at least 1 year since undergoing bariatric surgery, addressing a major research gap. The main themes that emerged from the interviews were motivators for bariatric surgery, barriers, and enablers for overcoming post-surgery challenges, and strategies and suggestions for post–bariatric surgery lifestyle changes and community-based programs.

**Figure 2:**
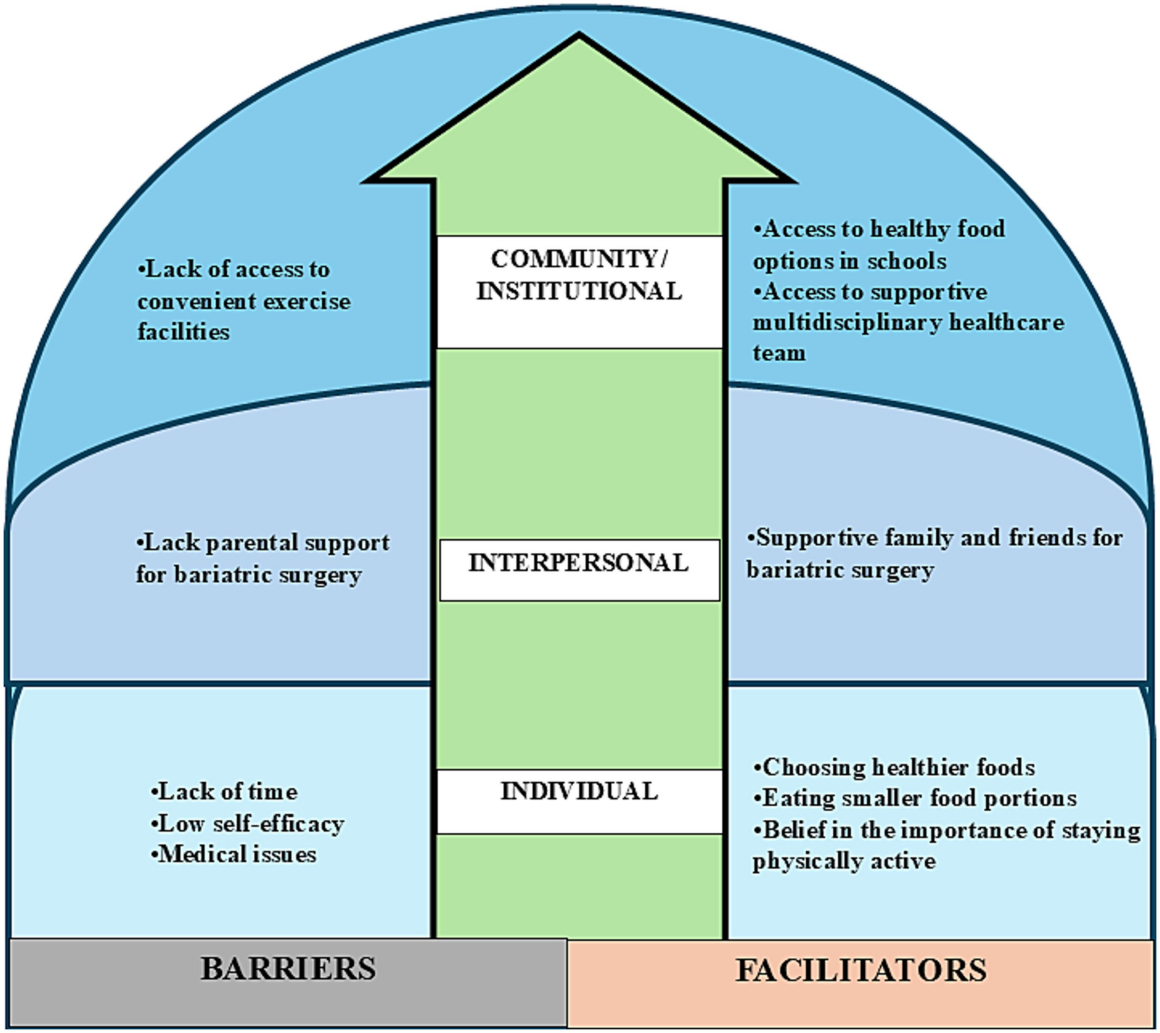
Barriers and facilitators of lifestyle factors among adolescents ≥1 year post–bariatric surgery, classified according to the social-ecological model.

According to Figure 2, the participants faced individual-level barriers such as time constraints due to academic commitments and a lack of self-efficacy, which affected their exercise and dietary habits. Medical issues such as dizziness also acted as obstacles. Meanwhile, interpersonal barriers included limited parental support for bariatric surgery. At the community level, the lack of accessible exercise facilities and supportive staff to train adolescents in using the equipment hindered regular physical activity. The findings of the study also highlight individual-level facilitators, such as making better food choices, maintaining regular physical activity, and recognizing its importance, which contribute to adopting a healthier lifestyle after surgery to achieve weight loss goals. At the interpersonal level, the support of family and friends significantly aided in motivating adolescents to undergo bariatric surgery. At the institutional level, the support of a multidisciplinary healthcare team was essential for meeting post-surgery challenges.

Childerhose et al. highlighted the significant influence of parents, particularly mothers, in the decisions of adolescents to undergo surgery (33). Müssener et al. found that adolescents with obesity were motivated by avoiding adverse outcomes, including shame, breathing difficulties, and social isolation, as well as improving their mental health (34). Peacock et al. investigated nonadherence to physical activity among patients who underwent bariatric surgery, identifying internal barriers such as motivation and biological issues and external barriers such as pain and postoperative problems (35). This contrasts with the findings of the current study, which identified barriers related to social, environmental, psychological, and medical factors, highlighting differences between adolescent and adult experiences, particularly with respect to timing and scheduling difficulties.

This study found that the main challenge encountered by participants was a lack of time, primarily due to substantial academic workload and responsibilities, which hindered their exercise routines. Likewise, Childerhose et al. identified heavy academic duties as a primary barrier for adolescents (33) and Foley et al. (36) highlighted the lack of time as a common barrier to promoting healthy eating and physical activity among adolescents. Moreover, Abdelghaffar et al. found that lack of time represented a significant barrier to physical activity among school-age adolescents, especially girls, influenced by factors such as heavy academic workloads, excessive screen time, socializing, poor time management skills, and household chores (37). This study further identified environmental, psychological, and medical barriers to regular exercise, such as poor gym skills, boredom, and medical conditions, which align with the findings of Nelson et al., who noted themes such as unfavorable weather, lack of motivation, and inadequate social support in adolescents (38).

The literature has identified insurance coverage as a barrier to bariatric surgery for adolescents, with Perez et al. highlighting its influence on access based on race and ethnicity (39). Armstrong et al. (2019) noted significant obstacles for low-income individuals, despite advocacy from the American Academy of Pediatrics (40). In contrast to previous studies, all participants in the present study had insurance coverage for their surgeries and consultations, which served as a motivator. In addition, most participants in this study reported minimal concerns about diet and physical activity, but encountered other challenges, such as dizziness, lethargy, and hair loss. Some of the participants also experienced stomach ulcers and hypoglycemia. Li et al. highlighted the participants’ awareness of severe and rapid hair loss after surgery (41). The participants in this study valued supportive care and guidance from healthcare providers, especially the management of dumping syndrome.

The findings highlight significant lifestyle changes after the surgery, including smaller food portions and adherence to healthier eating habits, such as reduced sugar. Despite these challenges, the participants reported engaging in regular physical activity, reflecting efforts to maintain their health after surgery, which is aligned with the findings of previous studies (41, 42).

At the institutional level, most participants sought weight loss assistance from a healthcare team, which consisted of doctors, dietitians, and nurses. They recognized that support from the healthcare team and personal management were both crucial in navigating the adjustments needed after bariatric surgery. Therefore, they expressed no need for the development of a new program. Müssener et al. highlighted the importance of psychosocial assistance to address relapse concerns and create new healthy lifestyle habits, roles, and identities (34). In this study, strategies for overcoming post-surgery challenges included seeking support from healthcare teams and developing self-management skills. Availability of healthy food options in schools and universities supported lifestyle changes, and this should be reinforced through the required local policies.

The participants in Li et al.’s study emphasized the importance of informed decision-making and realistic expectations for adolescents considering bariatric surgery (41). Although some did not consistently follow medical advice, such as taking vitamin supplements, they advised future bariatric surgery candidates to prioritize compliance. Likewise, Nordin et al. found that adolescents faced challenges navigating healthcare transitions post-surgery, with the participants expressing confusion within the healthcare system (42). In contrast, the participants in this study cited receiving adequate support and guidance from their healthcare team to deal with medical challenges after surgery. They highlighted the importance of healthcare support and adherence to medical instructions, including medication and nutritional supplements, as crucial for overcoming post-surgery challenges.

Although psychological assessment of the adolescents in this study before or after the surgery is not available, several participants raised body image-related issues, such as hair loss following the surgery. Previous research found that adolescents with severe obesity who underwent bariatric surgery had a high prevalence of psychiatric disorders before surgery, which persisted for at least 10 years postoperatively (43). Similarly, studies have shown that although adolescents achieve sustained weight loss after bariatric surgery, mental health issues persisted (44–46). These findings highlight the need for pre- and post-surgery psychological assessments as part of the care, which could help identify adolescents who require ongoing support.

This study recommends providing adolescents with education after bariatric surgery on alternative exercise options to address time constraints, engaging parents to influence dietary choices positively, and fostering effective time management skills. Collaboration between schools and healthcare facilities is also advised for universal promotion of healthy lifestyles. Thus, supportive collaborations should be maintained for improved post-surgery outcomes.

### 4.1 Strengths and limitations

This study is the first to provide insights into the post-surgery lifestyle experiences among adolescents in the UAE, a country with high prevalence of adolescent overweight and obesity. It is also one of the few studies investigating the lived experiences of adolescents who underwent bariatric surgery, including barriers and enablers to a healthy lifestyle. The main limitation of this study pertains to its small sample size, which only comprised 15 adolescents, predominantly females and recruited from a single healthcare facility. Thus, their views and lived experiences may not reflect the broader demographics of adolescents seeking bariatric surgery in the UAE. To address this limitation, future research should involve multiple healthcare settings and reach data saturation to broaden the relevance of the findings. Another major strength is the use of the SEM to explore the factors influencing dietary and physical activity behaviors, as well as related barriers and facilitators at various levels, that may affect adolescents’ lived experiences after bariatric surgery.

The study’s qualitative methods also restrict the generalizability of the findings, and the presence of parents during the interviews may have affected the participants’ willingness to disclose certain information in their responses. On the other hand, the presence of parents may have reassured adolescents and facilitated safe interaction with the facilitator, particularly given that all interviews were conducted online and neither the participants nor their families were previously acquainted with the facilitator.

## 5 Conclusion

The study highlights the impact of social factors on adolescents’ decisions and experiences with bariatric surgery in the UAE. The participants stressed the importance of ongoing healthcare support and adopted healthier habits, such as improved nutrition and portion control, after the surgery. However, they struggled to integrate regular exercise due to time constraints. Understanding these lifestyle changes is crucial for enhancing adolescent healthcare and ensuring future well-being. Continuous monitoring and interdisciplinary collaborations are vital for sustaining post-surgery outcomes and promoting overall health among adolescents undergoing bariatric surgery interventions.

## Data Availability

The original contributions presented in the study are included in the article/Supplementary material, further inquiries can be directed to the corresponding author.

## Supplementary material

Supplementary material S1: Interview Discussion Guide.

Supplementary material S2: Consolidated criteria for reporting qualitative studies (COREQ) Checklist.

## References

[1] World Health Organization, Overweight and obesity: Fact sheet (2025). Available online at: https://www.who.int/news-room/fact-sheets/detail/obesity-and-overweight

[2] Zhang X Liu J Ni Y Yi C Fang Y Ning Q . Global prevalence of overweight and obesity in children and adolescents: a systematic review and meta-analysis. JAMA Pediatr. (2024) 178:800–13. doi: 10.1001/jamapediatrics.2024.1576, PMID: 38856986 PMC11165417

[3] Badawi AEAE Almansoori HM Alnuaimi RA Howari FM. Factors influencing childhood and adolescent obesity in the Arab Gulf States: a systematic review. Glob J Health Sci. (2021) 13:98. doi: 10.5539/gjhs.v13n10p98

[4] Ng SW Zaghloul S Ali H Harrison G Yeatts K El Sadig M . Nutrition transition in the United Arab Emirates. Eur J Clin Nutr. (2011) 65:1328–37. doi: 10.1038/ejcn.2011.135, PMID: 21772317 PMC3304306

[5] Haroun D Mechli R Sahuri R AlKhatib S Obeid O El Mallah C . Metabolic syndrome among adolescents in Dubai, United Arab Emirates, is attributable to the high prevalence of low HDL levels: a cross-sectional study. BMC Public Health. (2018) 18:1284. doi: 10.1186/s12889-018-6215-x, PMID: 30463538 PMC6249919

[6] Abdelgadir E Rashid F Bashier A Zidan M Thalange N Hassanein M . Prevalence of overweight and obesity in children and adolescents: a large-scale population-based study. Diabetes Obes Metab. (2025) 27:4576–80. doi: 10.1111/dom.16449, PMID: 40345170

[7] Baniissa W Radwan H Rossiter R Fakhry R Al-Yateem N Al-Shujairi A . Prevalence and determinants of overweight/obesity among school-aged adolescents in the United Arab Emirates: a cross-sectional study of private and public schools. BMJ Open. (2020) 10:1–11. doi: 10.1136/bmjopen-2020-038667 PMC7735131 33310793

[8] Simmonds M Llewellyn A Owen CG Woolacott N. Predicting adult obesity from childhood obesity: a systematic review and meta-analysis. Obes Rev. (2016) 17:95–107. doi: 10.1111/obr.12334, PMID: 26696565

[9] Inge TH King WC Jenkins TM Courcoulas AP Mitsnefes M Flum DR . The effect of obesity in adolescence on adult health status. Pediatrics. (2013) 132:1098–104. doi: 10.1542/peds.2013-2185, PMID: 24249816 PMC3838536

[10] Oranika US Adeola OL Egbuchua TO Okobi OE Alrowaili DG Kajero A . The role of childhood obesity in early-onset type 2 diabetes mellitus: a scoping review. Cureus. (2023) 15:e48037. doi: 10.7759/cureus.48037, PMID: 38034219 PMC10687489

[11] Reece LJ Bissell P Copeland RJ. ‘I just don’t want to get bullied anymore, then I can lead a normal life’; insights into life as an obese adolescent and their views on obesity treatment. Health Expect. (2016) 19:897–907. doi: 10.1111/hex.12385, PMID: 27403849 PMC4989446

[12] Rupp K McCoy SM. Bullying perpetration and victimization among adolescents with overweight and obesity in a nationally representative sample. Child Obes. (2019) 15:323–30. doi: 10.1089/chi.2018.0233, PMID: 31062988 PMC7364321

[13] Valderas JM Starfield B Sibbald B Salisbury C Roland M. Defining comorbidity: implications for understanding health and health services. Ann Fam Med. (2009) 7:357–63. doi: 10.1370/afm.983, PMID: 19597174 PMC2713155

[14] Garcia VF. Adolescent bariatric surgery In: Surgical Management of Obesity, Philadelphia, PA: Elsevier (Saunders) (2007). 315–23. doi: 10.1016/B978-1-4160-0089-1.50042-X

[15] Steinbeck KS Lister NB Gow ML Baur LA. Treatment of adolescent obesity. Nat Rev Endocrinol. (2018) 14:331–44. doi: 10.1038/s41574-018-0002-8, PMID: 29654249

[16] Fitzgerald DA Baur L. Bariatric surgery for severely obese adolescents. Paediatr Respir Rev. (2014) 15:227–30. doi: 10.1016/j.prrv.2014.06.001, PMID: 25092494

[17] Calcaterra V Cena H Pelizzo G Porri D Regalbuto C Vinci F . Bariatric surgery in adolescents: to do or not to do? Children. (2021) 8:453. doi: 10.3390/children8060453, PMID: 34072065 PMC8204230

[18] Pratt JSA Browne A Browne NT Bruzoni M Cohen M Desai A . ASMBS pediatric metabolic and bariatric surgery guidelines, 2018. Surg Obes Relat Dis. (2018) 14:882–901. doi: 10.1016/j.soard.2018.03.019, PMID: 30077361 PMC6097871

[19] Beamish AJ Ryan Harper E Järvholm K Janson A Olbers T. Long-term outcomes following adolescent metabolic and bariatric surgery. J Clin Endocrinol Metab. (2023) 108:2184–92. doi: 10.1210/clinem/dgad155, PMID: 36947630 PMC10438888

[20] Sarwer DB Dilks RJ Spitzer JC Berkowitz RI Wadden TA Moore RH . Changes in dietary intake and eating behavior in adolescents after bariatric surgery: an ancillary study to the teen-LABS consortium. Obes Surg. (2017) 27:3082–91. doi: 10.1007/s11695-017-2764-9, PMID: 28625002 PMC5747929

[21] Elkhoury D Elkhoury C Gorantla VR. Improving access to child and adolescent weight loss surgery: a review of updated national and international practice guidelines. Cureus. (2023) 15:e38117–5. doi: 10.7759/cureus.38117, PMID: 37252536 PMC10212726

[22] Golden SD Earp JAL. Social ecological approaches to individuals and their contexts: twenty years of health education & behavior health promotion interventions. Health Educ Behav. (2012) 39:364–72. doi: 10.1177/1090198111418634, PMID: 22267868

[23] McLeroy KR Bibeau D Steckler A Glanz K. An ecological perspective on health promotion programs. Health Educ Q. (1988) 15:351–77. doi: 10.1177/109019818801500401, PMID: 3068205

[24] MMCE P CMP P HGDSM N. Describing studies on childhood obesity determinants by socio-ecological model level: a scoping review to identify gaps and provide guidance for future research. Int J Obes. (2019) 43:1883–90. doi: 10.1038/s41366-019-0411-3 31285521

[25] World Health Organization, in Adolescent health. Available online at: https://www.who.int/health-topics/adolescent-health (Accessed July 31, 2025).

[26] National Center for Health Statistics, in Growth charts (2024). Available online at: https://www.cdc.gov/growthcharts/ (Accessed: July 22, 2025)

[27] Hu D Zhou S Crowley-Mchattan ZJ Liu Z. Factors that influence participation in physical activity in school-aged children and adolescents: a systematic review from the social ecological model perspective. Int J Environ Res Public Health. (2021) 18:1–20. doi: 10.3390/ijerph18063147 PMC8003258 33803733

[28] Golden SD McLeroy KR Green LW Earp JAL Lieberman LD. Upending the social ecological model to guide health promotion efforts toward policy and environmental change. Health Educ Behav. (2015) 42:8S–14S. doi: 10.1177/1090198115575098, PMID: 25829123

[29] Braun V Clarke V. Using thematic analysis in psychology. Qual Res Psychol. (2006) 3:77–101. doi: 10.1191/1478088706qp063oa

[30] Pope C Mays N. Qualitative research: reaching the parts other methods cannot reach: an introduction to qualitative methods in health and health services research. BMJ. (1995) 311:42–5. doi: 10.1136/bmj.311.6996.42, PMID: 7613329 PMC2550091

[31] Williams M Moser T. The art of coding and thematic exploration in qualitative research. Int Manag Rev. (2019) 15:45–55.

[32] Tong A Sainsbury P Craig J. Consolidated criteria for reporting qualitative research (COREQ): a 32-item checklist for interviews and focus groups. Int J Qual Health Care. (2007) 19:349–57. doi: 10.1093/intqhc/mzm042, PMID: 17872937

[33] Childerhose JE Eneli I Steele KE. Adolescent bariatric surgery: a qualitative exploratory study of US patient perspectives. Clinical Obesity. (2018) 8:345–54. doi: 10.1111/cob.12272, PMID: 30107093

[34] Müssener U Örn M Olbers T Löf M Sjögren L. Adolescents’ and professionals’ experiences of metabolic and bariatric surgery and requirements for preoperative and postoperative support through mHealth: a qualitative study. BMJ Open. (2022) 12:11. doi: 10.1136/bmjopen-2022-064893, PMID: 36332966 PMC9639096

[35] Peacock JC Sloan SS Cripps B. A qualitative analysis of bariatric patients’ post-surgical barriers to exercise. Obes Surg. (2014) 24:292–8. doi: 10.1007/s11695-013-1088-7, PMID: 24092517

[36] Foley BC Mihrshahi S Shrewsbury VA Shah S. Adolescent-led strategies within the home to promote healthy eating and physical activity. Health Educ J. (2019) 78:138–48. doi: 10.1177/0017896918790295

[37] Abdelghaffar EA Hicham EK Siham B Samira EF Youness EA. Perspectives of adolescents, parents, and teachers on barriers and facilitators of physical activity among school-age adolescents: a qualitative analysis. Environ Health Prev Med. (2019) 24:21. doi: 10.1186/s12199-019-0775-y, PMID: 30961543 PMC6454728

[38] Nelson MC Kocos R Lytle LA Perry CL. Understanding the perceived determinants of weight-related behaviors in late adolescence: a qualitative analysis among college youth. J Nutr Educ Behav. (2009) 41:287–92. doi: 10.1016/j.jneb.2008.05.005, PMID: 19508935

[39] Perez NP Westfal ML Stapleton SM Stanford FC Griggs CL Pratt JS . Beyond insurance: race-based disparities in the use of metabolic and bariatric surgery for the management of severe pediatric obesity. Surg Obes Relat Dis. (2020) 16:414–9. doi: 10.1016/j.soard.2019.11.020, PMID: 31917198 PMC7058484

[40] Armstrong SC Bolling CF Michalsky MP Reichard KW. Pediatric metabolic and bariatric surgery: evidence, barriers, and best practices. Pediatrics. (2019) 144:e20193223. doi: 10.1542/peds.2019-3223, PMID: 31656225

[41] Li MK Sathiyamoorthy T Regina A Strom M Toulany A Hamilton J. ‘Your own pace, your own path’: perspectives of adolescents navigating life after bariatric surgery. Int J Obes. (2021) 45:2546–53. doi: 10.1038/s41366-021-00928-w, PMID: 34385587 PMC8359630

[42] Nordin K Brorsson AL Ekbom K. Adolescents’ experiences of obesity surgery: a qualitative study. Surg Obes Relat Dis. (2018) 14:1157–62. doi: 10.1016/j.soard.2018.04.003, PMID: 29903687

[43] Bruzé G Järvholm K Norrbäck M Ottosson J Näslund I Söderling J . Mental health from 5 years before to 10 years after bariatric surgery in adolescents with severe obesity: a Swedish nationwide cohort study with matched population controls. Lancet Child Adolesc Health. (2024) 8:135–46. doi: 10.1016/S2352-4642(23)00311-5, PMID: 38159575

[44] Järvholm K Karlsson J Olbers T Peltonen M Marcus C Dahlgren J . Characteristics of adolescents with poor mental health after bariatric surgery. Surg Obes Relat Dis. (2016) 12:882–90. doi: 10.1016/j.soard.2016.02.001, PMID: 27134198

[45] Järvholm K Bruzé G Peltonen M Marcus C Flodmark CE Henfridsson P . 5-year mental health and eating pattern outcomes following bariatric surgery in adolescents: a prospective cohort study. Lancet Child Adolesc Health. (2020) 4:210–9. doi: 10.1016/S2352-4642(20)30024-9, PMID: 31978372

[46] Tuli S Lopez Lopez AP Nimmala S Pedreira CC Singhal V Bredella MA . Two-year study on the impact of sleeve gastrectomy on depressive and anxiety symptoms in adolescents and young adults with moderate to severe obesity. Obes Surg. (2024) 34:568–75. doi: 10.1007/s11695-023-07025-z, PMID: 38177554

