# Supplementary material for: Navigating barriers and facilitators to lifestyle changes after bariatric surgery among Emirati adolescents: a qualitative study
Source: Front Public Health. 2025 Nov 26;13:1702340. doi: 10.3389/fpubh.2025.1702340 (PMC12689554; doi:10.3389/fpubh.2025.1702340)
Supplement: Supplementary material S1: — Interview Discussion Guide. [file Supplementary_file_1.docx]

**Exploring food and physical activity behaviors for adolescent post bariatric surgery: A qualitative Study**

**Interview questions**

**Introduction**

Thank you for agreeing to participate in the interviews we are conducting to understand more about the changes in food and physical activity behaviours of adolescents after bariatric surgery. Your comments will assist in the development of methods and activities to aid weight loss following bariatric surgery. The interview will last around 20- 30minutes. I am going to audio-tape our conversation so that we will be able to collect a complete information.

**Section 1. Personal Level:**

**Motivations:** First, we will discuss about your reasons for choosing bariatric surgery and how you manage your weight after the surgery:

1. What motivated you to go for bariatric surgery in the first place?

**Physical activity and dietary habits after bariatric surgery:** Now, I will ask you a few questions about your diet and physical activity after the surgery:

1. Some people believe bariatric surgery is a great way to reduce weight without exercising. What is your opinion?
2. Do you exercise regularly after bariatric surgery? Why? Why not?
3. Do you feel that your eating habits have changed after the surgery? In what way (s)?

**Section 2. Interpersonal Level**

**Challenges and Facilitators:** Let's speak about some of the issues you experienced following surgery to follow a special food pattern.

1. What were some of the challenges you faced about your food and physical activity after bariatric surgery?
2. How do you think these difficulties can be overcome (removed)?
3. What kind of assistance do you need to improve your weight-loss?
4. Who do you think can help you to overcome these difficulties?

**Section 3. Institutional Level (Healthcare Team) and Community Levels:**

1. If your healthcare team wants to develop a program to facilitate weight loss for adolescents who did bariatric surgery, what do you think this program should have?
2. What is your opinion of the foods available in your school/ college or place of work?
3. In your opinion, what are some of the factors that can support teenagers in overcoming challenges related to food, exercise, and weight after bariatric surgery?
4. If there is a special program that can help adolescents reach their healthy weight post-bariatric surgery, what would make you interested in joining this program?

**استكشاف السلوكيات الغذائية والنشاط البدني للمراهقين بعد جراحة السمنة:**

**دراسة نوعية**

**أسئلة المقابلة**

**مقدمة**

نشكرك على موافقتك على المشاركة في المقابلات التي نجريها لفهم المزيد حول التغييرات في سلوكيات الغذاء والنشاط البدني للمراهقين بعد جراحة علاج البدانة. ستساعد تعليقاتك في تطوير السلوكيات و التعليمات للمساعدة في إنقاص الوزن بعد جراحة علاج السمنة. ستستغرق المقابلة حوالي 20-30 دقيقة. سأقوم بتسجيل محادثتنا على شريط صوتي حتى نتمكن من جمع معلومات كاملة.

**القسم 1. المستوى الشخصي:**

**1- الدوافع:** أولاً ، سنناقش أسباب اختيارك لجراحة السمنة وكيف تدير وزنك بعد الجراحة:

ما الذي دفعك لإجراء جراحة السمنة في المقام الأول؟

**2 - النشاط البدني والعادات الغذائية بعد جراحة السمنة:** الآن سأطرح عليك بعض الأسئلة حول نظامك الغذائي والنشاط البدني بعد الجراحة:

1. يعتقد بعض الناس أن جراحة السمنة هي طريقة رائعة لإنقاص الوزن دون ممارسة الرياضة. ما هو رأيك؟
2. هل تمارس الرياضة بانتظام بعد جراحة السمنة؟ لماذا نعم ا؟ أو لماذا لا؟
3. هل تشعر أن عاداتك الغذائية قد تغيرت بعد الجراحة؟ كيف ؟

**القسم 2. المستوى الشخصي**

**التحديات والمُيسِّرات:** لنتحدث عن بعض المشكلات التي واجهتها بعد الجراحة لاتباع نمط غذائي خاص.

1. ما هي بعض التحديات التي واجهتها بشأن طعامك ونشاطك البدني بعد جراحة السمنة؟
2. كيف برأيك يمكن التغلب على (إزالة) هذه الصعوبات؟
3. ما نوع المساعدة التي تحتاجها لتحسين فقدان الوزن؟
4. من برأيك يمكنه مساعدتك في التغلب على هذه الصعوبات؟

**القسم 3. المستوى المؤسسي (فريق الرعاية الصحية):**

إذا كان فريق الرعاية الصحية الخاص بك يريد تطوير برنامج لتسهيل إنقاص الوزن للمراهقين الذين أجروا جراحة السمنة ، فماذا تعتقد أن هذا البرنامج يجب أن يحتوي عليه؟

**مستوى المجتمع المحلي:**

1. ما هو رأيك في الأطعمة المتوفرة في مدرستك / كليتك أو مكان عملك؟
2. ما هي برأيك بعض العوامل التي يمكن أن تساعد المراهقين في التغلب على التحديات المتعلقة بالغذاء والتمارين والوزن بعد جراحة السمنة؟
3. إذا كان هناك برنامج خاص يمكن أن يساعد المراهقين للوصول إلى وزن صحي بعد جراحة السمنة ، فما الذي يجعلك مهتمًا بالانضمام إلى هذا البرنامج؟
